# Comprehensive adulteration detection of sesame oil based on characteristic markers

**DOI:** 10.1016/j.fochx.2023.100745

**Published:** 2023-06-07

**Authors:** Zhe Chen, Jiashun Fu, Xinjing Dou, Zhuowen Deng, Xuefang Wang, Fei Ma, Li Yu, Yong-Huan Yun, Peiwu Li, Liangxiao Zhang

**Affiliations:** a School of Food Science and Engineering, Key Laboratory of Tropical Fruits and Vegetables Quality and Safety for State Market Regulation, Hainan University, Haikou 570228, China; b Key Laboratory of Biology and Genetic Improvement of Oil Crops, Ministry of Agriculture and Rural Affairs, Oil Crops Research Institute, Chinese Academy of Agricultural Sciences, Wuhan 430062, China; c College of Food Science and Engineering, Nanjing University of Finance and Economics/Collaborative Innovation Center for Modern Grain Circulation and Safety, Nanjing 210023, China; d Hubei Hongshan Laboratory, Wuhan 430070, China; e Xianghu Laboratory, Hangzhou 311231, China

**Keywords:** Sesame oil, Authentication detection, Characteristic markers, Elimination

## Abstract

Sesame oil has a unique flavor and is very popular in Asian countries, and this leads to frequent adulteration. In this study, comprehensive adulteration detection of sesame oil based on characteristic markers was developed. Initially, sixteen fatty acids, eight phytosterols, and four tocopherols were utilized to construct an adulteration detection model, which screened seven potentially adulterated samples. Subsequently, confirmatory conclusions were drawn based on the characteristic markers. Adulteration with rapeseed oil in 4 samples was confirmed using the characteristic marker of brassicasterol. The adulteration of soybean oil in 1 sample was confirmed using the isoflavone. The adulteration of 2 samples with cottonseed oil was demonstrated by sterculic acid and malvalic acid. The results showed that sesame oil adulteration could be detected by screening positive samples using chemometrics and verifying with characteristic markers. The comprehensive adulteration detection method could provide a system approach for market supervision of edible oils.

## Highlights

• Comprehensive adulteration detection of sesame oil based on markers was developed.
• The potential adulterated oils are first listed according their market prices.
• The characteristic markers of these cheaper oils are used to identify adulteration.

## 1 Introduction

Sesame oil, which is a precious oil produced from sesame seeds, possesses a characteristic flavor and various nutrients (Shi et al., 2018). In China, a high incidence of adulteration exists in sesame oil because sesame oil is more expensive (three to 10 times) than the price of common edible oils (Li et al., 2023, Seo et al., 2010, Zhang et al., 2017). Generally, cheaper oils, such as soybean oil, palm oil, and cottonseed oil, are used to adulterate sesame oil. Edible oil adulteration violates fundamental consumer rights (the right to correct information and to buy ‘‘value-for-money’’) and poses a serious threat to public health (Ulberth & Buchgraber, 2000). Therefore, sesame oil adulteration detection is very necessary.

In the last 20 years or more, various methods have been proposed to detect adulterated sesame oils by the blending of sesame oil with other vegetable oils. Generally, the adulteration methods of sesame oils can be divided into four categories. The first one is a detection technology based on conventional physical and chemical indicators including the refractive index, acid value, saponification value, and refraction coefficient (Aued-Pimentel et al., 2006, Ulberth and Buchgraber, 2000) that can all be changed by processing procedures. Therefore, there is no difference in these indicators between authentic and adulterated sesame oils after oil processing. The second is a rapid detection technology based on photoelectromagnetic and sensory signals, such as near infrared, Raman, fluorescence spectroscopy, terahertz, electronic nose, and an electronic tongue (Berghian-Grosan and Magdas, 2021, Chen et al., 2018, Hai and Wang, 2006, Luo et al., 2018, Shuai et al., 2014). The advantage of these types of methods are that they are fast and nondestructive, but the chemical implications are often unclear. These methods typically rely on establishing a prediction model using chemometrics to achieve authenticity identification. The third are analytical methods based on metabolomics that target the common components of edible oils, such as fatty acids, phytosterols, triglycerides, and volatile organic compounds (Kang et al., 2002, Lee et al., 2001, Shukla and Singh, 2005, Dou et al., 2023, Zhang et al., 2015). The advantages of these methods are good reproducibility and high sensitivity, but the conclusions obtained using modeling methods depend on the authenticity and representativeness of the selected samples. Moreover, the above detection techniques have difficulty providing confirmatory results for judicial identification.

The fourth category is detection technologies based on characteristic markers such as characteristic secondary metabolites and exogenous markers. Chloroplast barcoding regions are used as a target to obtain barcoding information for the major vegetable oil species and to quantitatively identify the botanical origin of plant oils (Bakre, Gadmale, Toche, & Gaikwad, 2015). This proposed method is capable of distinguishing among different types of vegetable oils and detecting a level of 1 % (w/w) of canola oil in olive oil. Surface enhanced Raman spectroscopy (SERS) with silver nanorod array substrates has been developed to detect capsaicin, a marker of gutter oil that is difficult to remove (Tian et al., 2018). By combining a liquid phase extraction method and the SERS detection strategy, a capsaicin concentration as low as 30 mg/L can be detected from inoculated corn oil samples. DNA in olives, sesame, and soybeans in milled seeds and oils has been extracted and tested using PCR to investigate its use in edible oil traceability (Vietina, Agrimonti, & Marmiroli, 2013). The results showed that this is a cost effective method for the efficient detection of adulteration in edible oils. This method can accurately identify the adulteration of cheap vegetable oil and realize the detection of multiple adulteration (Ma, Li, Zhang, Yu, & Zhang, 2015). However, the current studies have primarily focused on markers of one or several adulterated edible oils. This means that these methods can only detect one or several target adulterated oils in sesame oil but not directly answer whether the sesame oil is authentic or not.

In this study, taking sesame oil as an example, a comprehensive adulteration detection of edible oil is proposed to systematically detect the characteristic markers of cheaper oils. The potential adulterated oils are first listed according their market prices. The characteristic markers of these cheaper oils are then detected and used to verified the authenticity of sesame oil.

## 2 Materials and methods

### 2.1 Materials and chemicals

The sesame oil, rapeseed oil, and soybean oil were purchased from local markets (Wuhan, Hubei, China). Before being used for analysis, all samples were stored in the dark. The standard samples of fatty acid methyl ester (FAME), brassicasterol, ergosterol, campesterol, campestanol, stigmasterol, β-sitosterol, Δ5-avenasterol, cycloartanol, cycloartenol, 24-methylene-cycloartenol, and cholestanol were purchased from the Sigma-Aldrich Chemical Co. (St. Louis, MO, USA). Daidzin, daidzein, genistin, and genistein were purchased from the Shanghai Yuanye Biotechnology Co. (Shanghai, China). Methyl sterculate (C_20_H_36_O_2_, Purity ≥ 98 %) and methyl malvalate (C_19_H_34_O_2_, purity ≥ 95 %) were purchased from the Matreya Lipid and Biochemistry Technology Co., Ltd., USA. All solvents used were of analytical grade, and all standards were stored at −20 °C in darkness.

### 2.2 Methods and statistical analysis

#### 2.2.1 Determination of fatty acid composition

Fatty acid composition is analyzed by the gas chromatography (GC) and gas chromatography mass spectrometer (GC–MS) according to the previous studies (Piravi-Vanak et al., 2009, Raes et al., 2003, Qian et al., 2013). Weigh 60 mg of oil sample accurately, add 2 mL of a mixture of ether and petroleum ether with a volume ratio of 1:1 and vortex; then add 2 mL of 2 M potassium hydroxide methanol solution, let the reaction stand for 2 h and then vortex, let it stand for 30 min. Add 2 mL of distilled water, let stand and partition; take 50 uL of the upper organic phase and dilute to 1 mL with petroleum ether..

GC: DB-23 column (30 m × 0.25 mm × 0.25 µm); carrier gas: 180 mL/min nitrogen, 30 mL/min hydrogen; 400 mL/min air; 1 µL injection volume; 150:1 splitting ratio; 250 °C gasification temperature; 280 °C detector temperature; column initial temperature of 180 °C, maintained for 2 min. The initial temperature of the column was 180 °C and held for 2 min, then ramped up to 230 °C at 3 °C/min and held for 12 min, followed by ramping up to 224 °C at 2 °C/min and held for 0.2 min.

GC–MS: DB-23 column (30 m × 0.25 mm × 0.25 µm); partition ratio 20:1; gasification temperature 220 °C, column initial temperature 100 °C, hold for 2 min, ramp up to 215 °C with 10 °C/min program, hold for 0.1 min; then ramp up to 224 °C with 2 °C/min, hold for 2 min; carrier gas is high purity helium; column flow rate 1.2 mL/min; injection volume 1 uL. EI source; electron energy: 70 EV. Ion source temperature: 230 °C; detector temperature: 150 °C; solvent delay: 3 min; scanning mode is selective ion mode, selected ions: 55,67,74,79. The composition data were taken from area percent data without the use of correction factors and rounded off to the two decimal place.

#### 2.2.2 Determination of phytosterols by GC–MS

The phytosterols in edible oil were determined by GC–MS. The pretreatment and instrumental conditions were according to the previous study (Yang et al., 2019). The brassicasterol, ergosterol, campesterol and other 10 kinds of sterol standards, take a certain amount of dissolved in acetone solution, prepared in a brown bottle into 1 mg/mL standard solution, stored in −20 °C in the refrigerator for backup. The cholestanol standard solution was used as an internal standard solution. A 50 mg of oil was placed in a sealable tube with a 200 µL internal standard solution. Alkaline hydrolysis was performed by adding 5 mL of 2 M KOH in ethanol, and the mixture was shaken and heated at 75 °C for 30 min. After saponification, the tube was cooled to room temperature. Then, add 2 mL distilled water and 5 mL *n*-hexane, shake thoroughly and extract the unsaponifiable substance. The unsaponifiable substances were extracted three times with 3 mL *n*-hexane. Then, the *n*-hexane extracts are combined and blown dry with nitrogen. 100 µL *N*-methyl-*N*-trimethylsilylheptaflfluorobutyramide-1-methyl imidazole (95:5, v/v) mixture was added. Afterward, the vial was sealed and heated at 75 °C for 20 min, then cooled to room temperature. Dilute to 1 mL with *n*-hexane before GC–MS analysis. Phytosterols were identified by comparing their mass spectra and retention times with the reference standards. The phytosterol contents were calculated based on the standard curves obtained by the internal standard method with Cholestane alcohol as the internal standard. Each oil sample was tested three times and all results were recorded by mean value and standard deviation.

#### 2.2.3 Tocopherol determination

The vitamin E content of the samples was determined by the method described in the references (Goffman & Böhme, 2001). Add 5.0 g of vegetable oil (accurate to 0.01 g) with 10 mL of hexane, mixed evenly and placed in an ultrasonic oscillator, let to 50 mL with a brown volume flask to 50 mL, filtered with 0.45 um filter membrane and measured by the liquid phase. The concentration gradients of 1 µg/mL, 10 µg/mL, 20 µg/mL, 40 µg/mL, 80 µg/mL, and 100 µg/mL were prepared with methanol respectively α-tocopherol, δ-tocopherol, β-tocopherol, γ-tocopherol Mixed standard working solution of tocopherol and stored under −20 °C away from light. The chromatographic conditions for drawing the standard curve are as follows: UV detector wavelength 294 nm, mobile phase methanol, flow rate 0.9 mL/min, column temperature 40 °C, injection volume 10 µL. Each sample was tested three times, and all results were recorded as the average value. Tocopherol was identified by comparing the retention time with the corresponding standard. The content of tocopherol in the sample was treated by peak area normalization.

#### 2.2.4 UPLC-MS/MS method for isoflavones

5 g (accurate to 0.01 g) of edible oil was accurately weighed into a 10 mL centrifuge tube, and 5 mL *n*-hexane was added for 1 min vortex mixing and stored at 4 °C for later use. The SPE column was activated with 5 mL methanol and 5 mL *n*-hexane successively at a flow rate of 1 mL /min. The liquid to be purified was put into the SPE column, rinsed and discarded with 5 mL 10 % ethyl acetate + *n*-hexane. 3 mL methanol was added to the SPE column, and the eluent was collected in a 10 mL centrifuge tube. The eluent was placed on a nitrogen blower, the water bath temperature was 50 °C, and the nitrogen was blown to dry. The solution was re-dissolved with 0.5 mL of 50 % methanol + water, vortexed for 30 s and filtered through a 0.22 μm filter membrane. The eluent was determined by liquid chromatography mass spectrometer. The standard solutions were prepared as follows: Accurately weigh 10 mg of daidzein, daidzein, genistein and genistein, respectively, and fill them in 10 mL brown volumetric flask with methanol. Standard working solutions with concentration gradients of 0.50 µg/L, 1.00 µg/L, 5.00 µg/L, 10.00 µg/L, 20.00 µg/L, and 50.00 µg/L were respectively configured and stored under −20 °C, protected from light.

Isoflavones were analyzed by an UPLC-MS/MS (8050 Shimadzu Corp, Kyoto, Japan). The chromatographic column C18 (100 mm × 2.1 mm × 3.0 µm) was obtained from Waters. Mobile phase A was 0.01 % formic acid in the water, and mobile phase B was acetonitrile solution. The fellow rate was 0.2 mL/min, and the gradient program was as follows: solvent B was increased from 10 % to 85 % in 11 min, held at 85 % B for 0.5 min, then phase B was decreased to 10 % within 0.1 min and maintained for 3 min. The injection volume was 10 µL. MS data were collected in positive ionization mode by MRM mode equipped with electrospray ion source. The auxiliary gas collision gas is high purity nitrogen. The parameters such as spray voltage collision energy were optimized to the optimal sensitivity and quantified by the external standard method. The samples were collected twice, and the absolute difference in the determination results was less than 10 % of the arithmetic average value.

#### 2.2.5 Detection of characteristic fatty acids in cottonseed oil

Accurately weigh 0.1 g of edible oil into a 20 mL centrifuge tube. Then, add 10 mL of hexane and vortex for 1 min. Next, pipette 3 mL of the solution to be measured into the centrifuge tube and add 2 mL of KOH solution. Vortex the mixture for 7 min and carefully collect the supernatant. Proceed to add anhydrous sodium sulfate and filter the mixture using a membrane. Transfer the filtrate to a 1 mL injection vial and allow it to await analysis by GC–MS. Accurately weigh 25 mg of methyl steroid and 5 mg of methyl malvalate in a 10 mL volumetric flask, dissolve them in chromatographic grade *n*-hexane and fix the volume to the moment. Standard solution preparation: 25 mg of methyl sterculate and 25 mg of methyl malvalate are accurately weighed into a 10 mL volumetric flask, dissolved in chromatographic grade *n*-hexane and allowed to equilibrate to volume, shake well, as a stock solution, concentration of 2.5 mg/mL and 0.5 mg/mL, respectively, placed in the refrigerator stored at −18 °C. Temporary advance Line, respectively, absorb the standard solution 2.0, 4.0, 6.0, 8.0, 10.0 mL in a 10 mL volumetric flask, *n*-hexane volume.

Chromatographic conditions: DB-5 MS chromatographic column (60 m × 0.25 mm × 0.25 µm); High purity helium gas, flow rate of 1.0 mL/min; Temperature programming: the column temperature is 60 °C for 1 min, 10 °C/min to 180 °C, 4 °C/min to 230 °C, 20 °C/min. Rise to 280 °C for 2 min; The sample volume is 1 µL, and the sample is not divided into samples; The inlet temperature is 280 °C. Mass spectrometry conditions: ionization mode is electron bombardment. Ion source mode (EI), the ionization energy of 70 EV, ion source temperature of 250 °C, scan mode: full scan, scanning range of *m*/*z* 40 ∼ 550, solvent delay 7 min.

Multiple peaks could be seen from the total ion current chromatogram (TIC). The NIST 2008 spectral database was used for spectral database retrieval. Malvalic acid and sterculic acid were analyzed qualitatively and quantitatively by combining the retention time of the reference standard.

#### 2.2.6 Data processing

Data processing and statistical analysis were performed on MetaboAnalyst 4.0 platform (Chong, Wishart, & Xia, 2019).

## 3 Results and discussion

### 3.1 Potential adulterated oils based on their prices

**Fig. 1:**
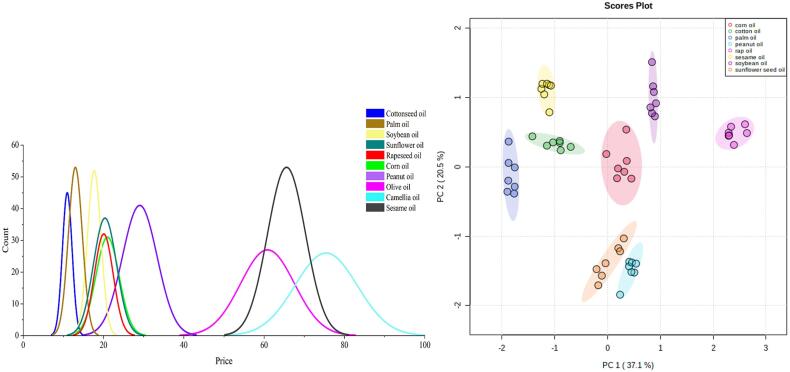
PCA model of eight pure vegetable oils with price as guidance. (a) Peak map of ten vegetable oils based on price distribution; (b) PCA model of eight vegetable oils screened by price.

Adulteration, which is generally economically motivated, is done by adding cheaper vegetable oils into an expensive oil (Cserháti, Forgács, Deyl, & Miksik, 2005). According to an investigation of the market prices of common edible oils, the price distribution of edible oils is shown in Fig. 1(a). Among the common edible oils, the prices of sesame oil, olive oil, and camellia oil are generally higher. Therefore, these edible oils possess the most serious adulteration problems (Pan et al., 2021, Shi et al., 2021). The prices of cottonseed oil and palm oil were much lower than others, and the prices of soybean oil, corn oil, peanut oil, rapeseed oil, and sunflower seed oil were at moderate levels. Based on economically motivated adulteration, the possibilities of camellia oil and olive oil to be adulterated into sesame oil were low. Based on the adulteration process of illegal traders, the difficulty of obtaining raw materials, and raw material production, it was concluded that palm oil, cottonseed oil, soybean oil, canola oil, and corn oil would be typically used as adulterated oils.

### 3.2 Authenticity identification using fatty acids, phytosterols, and tocopherols

Fatty acids are the most important components in edible oil. In addition, fatty acids are also the characteristic information of vegetable oils. The fatty acid composition of the eight target adulterant oils are shown in Table 1. In addition, small amounts of unsaponifiable components of vegetable oils, including various hydrocarbons, triterpenoids, carotenoids, tocopherols, and sterols, have been used by practitioners as characteristic components for screening adulterated vegetable oils (Bakre et al., 2015, Deme et al., 2021, Xu et al., 2018).

**Table 1:** Fatty acid composition of 8 kinds of edible oils.

Fatty acid	Myristic acid	Palmitic acid	Stearic acid	Oleic acid	Linoleic acid	Linolenic acid	Arachidic acid	Arachidonic acid	Behenic acid	Erucic acid	Malvalic acid	Sterculic acid
Sesame oil	ND ∼ 0.1	7.9 ∼ 12.0	4.5 ∼ 6.7	34.4 ∼ 45.5	36.9 ∼ 47.9	0.2 ∼ 1.0	0.3 ∼ 0.7	ND ∼ 0.3	ND ∼ 1.1	ND	ND	ND
Soybean oil	ND ∼ 0.2	8.0 ∼ 13.5	2.5 ∼ 5.4	17.7 ∼ 28.0	49.8 ∼ 59.0	5.0 ∼ 11.0	0.1 ∼ 0.6	ND ∼ 0.5	ND ∼ 0.7	ND	ND	ND
Cottonseed oil	0.6 ∼ 1.0	21.4 ∼ 26.4	2.1 ∼ 3.3	14.7 ∼ 21.7	46.7 ∼ 58.2	ND ∼ 0.7	0.2 ∼ 0.5	0.1 ∼ 0.8	ND ∼ 0.6	ND	0.68 ∼ 0.73	0.35 ∼ 0.39
Peanut oil	ND ∼ 0.1	8.0 ∼ 14.0	1.0 ∼ 4.5	35.0 ∼ 69.0	13.0 ∼ 43.0	ND ∼ 0.3	1.0 ∼ 2.0	0.2 ∼ 0.6	1.5 ∼ 4.5	ND	ND	ND
Corn oil	ND ∼ 0.3	8.6 ∼ 16.5	ND ∼ 3.3	20.0 ∼ 42.2	34.0 ∼ 65.6	ND ∼ 2.0	0.3 ∼ 1.0	ND ∼ 0.4	ND ∼ 0.1	ND	ND	ND
Sunflower oil	ND ∼ 0.2	5.0 ∼ 7.6	2.7 ∼ 6.5	14.0 ∼ 34.9	48.3 ∼ 47.0	ND ∼ 0.3	0.1 ∼ 0.5	ND ∼ 0.3	0.3 ∼ 1.5	ND	ND	ND
Palm oil	0.5 ∼ 2.0	39.3 ∼ 47.5	3.5 ∼ 6.0	36.0 ∼ 44.0	9.0 ∼ 12.0	ND ∼ 0.5	ND ∼ 1.0	ND ∼ 0.4	ND ∼ 0.2	ND	ND	ND
Common rapeseed oil	–	1.5 ∼ 6.0	–	8.0 ∼ 65.0	9.5 ∼ 30.0	5.0 ∼ 13.0	ND ∼ 3.0	3.0 ∼ 15.0	ND ∼ 2.0	3.0 ∼ 60.0	ND	ND
canola oil	–	2.5 ∼ 7.0	–	51.0 ∼ 70.0	15.0 ∼ 30.0	5.0 ∼ 14.0	0.2 ∼ 1.2	0.1 ∼ 4.3	ND ∼ 0.6	ND ∼ 3.0	ND	ND

In this study, fatty acids, phytosterols, and tocopherols of eight types of vegetable oils were fused to build an adulteration detection model. A principal component analysis (PCA) was used to analyze the chemical differences of the eight types of edible oils. As shown in Fig. 1(b), the fatty acids, phytosterols, and tocopherols of these oils were significantly different, and therefore provided a reference for the authenticity identification of sesame oil. In detail, the sesame oils were primarily composed of palm acid, palmitoleic acid, stearic acid, oleic acid, linoleic acid, linolenic acid, arachidonic acid, and behenic acid. Among them, oleic acid and linoleic acid had the highest contents, which is consistent with the findings of a previous study (Uzun, Ülger, & Çağirgan, 2002). The proportion of unsaturated fatty acids in the samples was 81.9–89.0 %, and this basically agreed with the range reported in a previous study (Özdemİr, Karaoğlu, Dağ, & Bekİroğlu, 2018). Moreover, phytosterols and tocopherols are not only important nutrients in edible oils, but they have also been used as important indicators for the authentication of sesame oils in previous reports (Xing et al., 2019, Xu et al., 2018). As shown in Table 2, the sesame oil primarily contained campesterol, stigmasterol, β-sitosterol, Δ5-avenasterol, cycloartanol, cycloartenol, and 24-methylene-cycloartanol, and did not have brassicasterol (Arab et al., 2022). Furthermore, the tocopherols in the sesame oils were primarily composed of γ-tocopherol (0.22–0.47 mg/g), and the total tocopherol content ranged from 0.22 to 0.59 mg/g.

**Table 2:** Characteristic component of 7 positive samples.

	Compounds	Sesame oils	Positive sample 1	Positive sample 2	Positive sample 3	Positive sample 4	Positive sample 5	Positive sample 6	Positive sample 7
Phytosterols (mg/kg)	Brassicasterol	ND	69.04	14.86	ND	37.27	62.70	ND	ND
	Campesterol	129.94 ± 36.00	326.57	213.31	138.90	231.06	318.80	120.89	90.5
	Stigmasterol	59.01 ± 19.75	32.13	97.02	69.61	62.93	114.96	53.702	47.803
	β- Sitosterol	515.86 ± 175.04	673.38	729.16	493.15	563.83	950.30	445.256	499
	Δ5-avenasterol	125.84 ± 39.92	77.95	140.97	113.97	79.57	139.02	103.13	111.18
	Cycloartanol	13.40 ± 5.35	7.67	17.84	11.49	14.28	27.07	12.454	8.67
	Cycloxylenol	26.09 ± 11.93	21.65	43.73	21.35	21.21	20.48	15.09	14.93
	24-methylene cycloxylanol	28.93 ± 51.64	13.56	242.23	9.29	11.15	38.99	20.642	25.02
Fatty acids (%)	Palmitic acid	9.17 ± 0.60	6.27	9.39	10.06	8.74	9.81	10.25	9.04
	Linolenic acid	0.56 ± 0.73	4.10	1.24	3.00	4.90	1.90	0.80	0.60
	Arachidonic acid	0.13 ± 0.07	0.04	0.08	0.06	0.08	0.09	0.00	0.02
	Tetracosenoic acid	0.11 ± 0.03	0.12	0.04	0.00	0.14	0.06	0.16	0.09
	Octadecadienoic acid	0.07 ± 0.02	0.05	0.09	0.08	0.11	0.13	0.10	0.05
	Linoleic acid	44.70 ± 2.67	27.87	42.80	47.60	41.76	37.78	49.87	45.98
	Pentadecaenoic acid	0.01 ± 0.01	0.02	0.01	0.01	0.01	0.01	0.01	0.01
	Eicosenoic acid	0.56 ± 0.11	0.11	0.22	0.26	0.23	0.19	0.17	0.16
	Oleic acid	38.94 ± 2.70	56.60	39.96	32.94	39.00	44.73	33.94	37.57
	Tricosadienoic acid	0.03 ± 0.02	0.04	0.01	0.03	0.07	0.06	0.03	0.01
Tocopherols (mg/kg)	α-tocopherol	8.57 ± 2.89	9.34	15.39	10.26	16.21	17.66	10.59	8.91
	β-tocopherol	0.31 ± 0.70	0.41	0.50	0.33	0.99	0.71	0.29	0.33
	γ-tocopherol	477.03 ± 185.04	466.33	433.10	681.93	460.51	508.28	336.81	474.76
	δ-tocopherol	16.8 ± 5.03	11.07	16.91	20.19	14.31	13.68	15.81	10.56
Markers of cottonseed oil (%)	Malvalic acid	ND	ND	ND	ND	ND	ND	0.01	0.02
	Sterculic acid	ND	ND	ND	ND	ND	ND	ND	0.09
Markers of soybean oil (ug/kg)	Daidzin	ND	ND	ND	0.21	ND	ND	ND	ND
	Genistin	ND	ND	ND	0.87	ND	ND	ND	ND

**Fig. 2:**
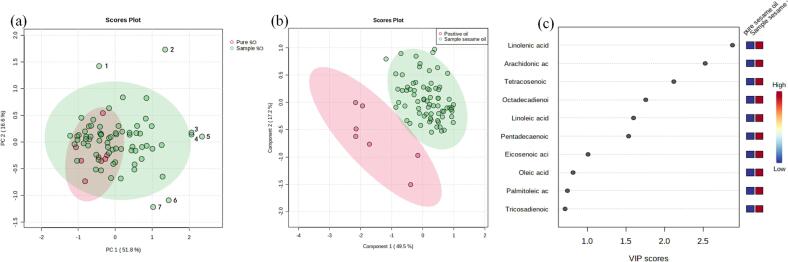
Screening of positive samples based on PLS-DA model. (a) shows PLS-DA of fatty acids, sterols and tocopherols content of sample sesame oil and pure sesame oil. Numbers in figures represent labeled positive sesame oil 1 to 7. (a) Shows Important measurement of PLS-DA of pure sesame oil and sesame oil samples: variable importance (VIP) in projection.The colored boxes on the right indicate the relative concentrations of the corresponding metabolite in each group under study. Sample codes: Pure so: Pure sesame oil; Sample so: Sample sesame oil; Te acid: Tetracosenoic acid; Oc acid:Octadecadienoic acid; Pe acid:Pentadecaenoic acid; Tr acid:Tricosadienoic acid. (c) Shows PLS-DA of 7 positive samples was determined by using 7 characteristic compounds as indicators.

Sixteen fatty acids, eight phytosterols, and four tocopherols were then used to build an adulteration detection model. Nine samples of pure sesame oil with confirmed attributes were selected, and PCA was used to establish a classification model for the pure and sample sesame oils (Fig. 2(a)). The sesame oil samples (green circles) and pure sesame oil (red triangles) overlapped severely and could not be separated, and the two principal components accounted for 68.4 % of the total variance, indicating that most of the sesame oil samples were real samples. It was obvious that seven of the 81 sesame oil samples were discrete samples. A partial least squares discriminant analysis model was then established to classify these seven discrete samples with other samples. To investigate whether the seven positive samples were significantly different from the remaining 74 sesame oils, partial least squares discriminant analysis (PLS-DA) was performed on the two groups of samples using the experimental data described above (Fig. 2(b)). As shown in Fig. 2(c), the variable importance in the projection (VIP) values of two compounds (linolenic acid and brassicasterol) were greater than two and those of seven compounds were greater than one. The cross-validation results indicated that the R^2^ and Q^2^ of the model were greater than 0.70. Thus, seven samples were identified as candidates of adulterated oils for subsequent validation.

### 3.3 Preliminary screening technology of sesame oil based on the fatty acid profiles

In the aforementioned experiments, the PLS-DA model established by analyzing fatty acids, sterols, and tocopherols was used to screen seven candidate oils out of 81 sesame oils. To ensure the accuracy of the results, a feature marker-based detection technique was subsequently employed to effectively address the limitations of this method and enhance the interpretability of the findings. Since fatty acids are the primary components of edible oil, they are difficult to eliminate using physical or chemical processing. Hence, fatty acids are commonly used to detect adulteration, and they can be used as not only fingerprints as in the above section, but also characteristic makers.

**Fig. 3:**
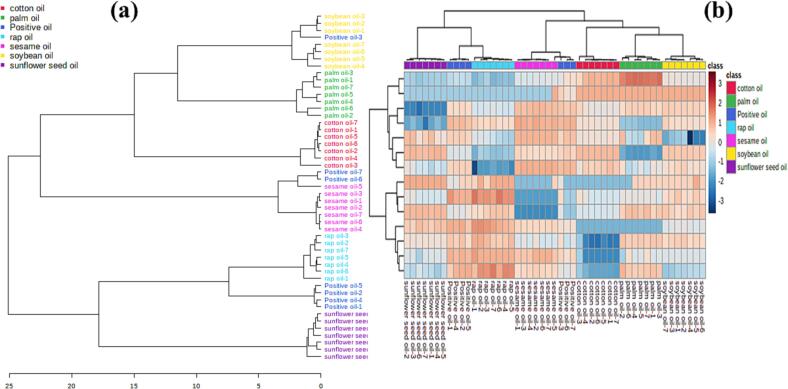
Clustering model of seven positive samples. (a) Shows hierarchical clustering dendrogram of seven positive samples and pure vegetable oil. (b) Shows hierarchical clustering heatmaps of seven positive samples and pure vegetable oil.

To verify the accuracy of the results, the fatty acid compositions of the 81 sesame oils are illustrated in Table 2. As shown in Table 2, positive samples 1–5 had high contents of linolenic acid, and were therefore identified as samples adulterated with rapeseed oil or soybean oil. This result was consistent with a previous study (Mahasti Shotorbani, Hamedi, Zandi, & Fahimdanesh, 2018). Moreover, hierarchical clustering analysis and heat maps were used to analyze the similarities of fatty acid composition of 81 edible oils. As shown in Fig. 3(a), seven positive samples were divided into three categories. Positive sample 3 was the most similar with soybean oils, while positive samples 1, 2, 4, and 5 were the most similar with rapeseed oils. However, positive samples 6 and 7 were the most similar with sesame oils. As illustrated in Table 2, the relative contents of linoleic acid in positive samples 6 and 7 were higher than the ones of the authentic sesame oils, while the relative contents of oleic acid in positive samples 6 and 7 were lower than in authentic sesame oils. The above results indicated that these two sesame oils might be adulterated with cottonseed oil or corn oil.

### 3.4 Confirmatory validation based on characteristic markers

#### 3.4.1 Validation of adulteration with cottonseed oil based on characteristic fatty acids

To verify the adulteration of the positive samples 1–7, the samples were subjected to verification of the characteristic markers of soybean oil, rapeseed oil, and cottonseed oil to determine the adulteration of the positive samples. As reported in a previous study (Aued-Pimentel, Lago, Chaves, & Kumagai, 2004), cottonseed oil is an oil extracted from Malva cotton and contains two unique fatty acids, malvalic acid and sterculic acid, and these have not been reported in sesame oil. Moreover, the two fatty acids contents in cottonseed oil are stable, which meets the requirements of serving as characteristic markers of adulterated cottonseed oil in sesame oil. By employing the highly sensitive detection technique of gas chromatography mass spectrometry (GC–MS), the presence of two fatty acids was detected in commercially available cottonseed oil, and the equivalent chain lengths and relative contents of the two characteristic fatty acids were calculated. Both markers were detected in the cottonseed oil samples. The feasibility of identifying adulterated cottonseed oil in edible oil was conducted based on characteristic markers that included malvalic acid and sterculic acid (Aued-Pimentel et al., 2004). In this study, the equivalent chain length of two characteristic markers and the retrieval of the mass spectrometry library were used to detect seven positive samples. The results (Table 2) showed that two target compounds were detected in positive samples 6 and 7. Therefore, positive samples 6 and 7 were identified as sesame oil adulterated with cottonseed oil.

#### 3.4.2 Detection of adulterated sesame oil with rapeseed oil based on characteristic phytosterol

As can be seen from Table 2, positive samples 1, 2, 4, and 5 contained brassicasterol, which is a characteristic marker of rapeseed oil. Moreover, compared with the authentic sesame oils, positive samples 1, 2, 4, and 5 had high contents of campesterol, β-sitosterol, 24-methylene cycloartenol, and the total content of phytosterols. These results indicated that positive samples 1, 2, 4, and 5 were identified as sesame oils adulterated with rapeseed oil. During the screening process for blended rapeseed oil in sesame seed oil, positive samples 1, 2, 4, and 5 were ultimately identified as adulterated oils. This method has strong universality, high sensitivity, a low detection limit, and has good application value for practical detection work.

Moreover, brassicasterol was not detected in positive sample 3, indicating positive sample 3 was not adulterated with rapeseed oil. The contents of β-sitosterol, 24-methylene cyclosterol, and the total content of phytosterols decreased in positive sample 3 but not in authentic sesame oils, indicating that this sample was adulterated with soybean oil.

#### 3.4.3 High-sensitive detection technique based on soybean markers

According to the above analysis, the fatty acid composition and phytosterol contents of positive sample 3 indicated that this sample was adulterated with soybean oil. Moreover, the content of γ-tocopherol of this sample was greater than the that in the sesame oils, verifying the same conclusion. To verify whether the positive sample 3 was adulterated with soybean oil, solid-phase extraction UPLC-MS/MS was used to detect isoflavones in positive sample 3. The determination results are shown in Table 2. Daidzein and genistein were detected in sample 3. This method has the advantages of a simple sample pretreatment, less consumption of organic reagents, short time, and can meet the needs of the qualitative and quantitative detection of isoflavones.

A determination method of isoflavones in vegetable oil was established by UPLC-MS/MS using the purification method of solid phase extraction and by optimizing the pretreatment steps of samples. The isoflavones, a unique component of soybean oil, were successfully detected from the sample, and this proved that positive sample 3 was adulterated with soybean oil.

## 4 Conclusion

Sesame oil adulteration with cheaper vegetable oils is a form of food fraud. The fatty acid composition, phytosterols, and tocopherols were used as fingerprints to detect sesame oil adulteration. Moreover, the adulteration detection of edible oil was more effective based on characteristic markers. However, the current studies have primarily focused on markers of one or several adulterant edible oils. This means that these methods can only detect one or several target adulterant oils in sesame oil, but not directly answer whether that sesame oil is authentic or not.

In this study, the potential adulterant oils were listed according their prices in market. PCA and PLS-DA models were established using fatty acids, phytosterols, and tocopherols to screen for positive samples. α-linolenic acid was then used to identify positive samples 1–5 that were adulterated with soybean oil and/or rapeseed oil. To verify whether the positive samples were adulterated, a characteristic marker of rapeseed (brassicasterol) was identified in the positive samples 1, 2, 4, and 5 and labeled as sesame oils adulterated with rapeseed oil. Moreover, UPLC-MS/MS was used to detect isoflavones in the edible oils. As a result, isoflavones were detected in positive sample 3. The adulteration of positive samples 6 and 7 with cottonseed oil was verified due to the presence of malvalic acid and sterculic acid. According to an analysis of fatty acids, phytosterols, isoflavones, and other characteristic markers, a comprehensive sesame oil adulteration detection method was developed based on the characteristic markers. It is worth mentioning that the method used in this study is suitable not only for sesame oil identification, but also for the identification of adulterated soybean oil, rapeseed oil, cottonseed oil, palm oil, and other target oils. This is of great significance for law enforcement departments to investigate and deal with the production and sale of counterfeit oils, regulate the sesame oil market, and safeguard the legitimate rights and interests of consumers.

## CRediT authorship contribution statement

**Zhe Chen:** Methodology, Writing – original draft, Writing – review & editing. **Jiashun Fu:** Methodology. **Xinjing Dou:** Methodology. **Zhuowen Deng:** Formal analysis. **Xuefang Wang:** Investigation. **Fei Ma:** Formal analysis. **Li Yu:** Formal analysis. **Yong-Huan Yun:** Writing – review & editing. **Peiwu Li:** Supervision, Funding acquisition. **Liangxiao Zhang:** Conceptualization, Writing – original draft, Writing – review & editing, Funding acquisition.

## Declaration of Competing Interest

The authors declare that they have no known competing financial interests or personal relationships that could have appeared to influence the work reported in this paper.

## Data Availability

Data will be made available on request.
